# Sleeping Away: The Hidden Toll of Self‐reported Sleep Health and Dementia in Kenyan Adults with Chronic Diseases

**DOI:** 10.1002/alz70855_106852

**Published:** 2025-12-24

**Authors:** Catherine Ajalo, David Andai, Jasmit Shah, Anne Nyambura Njogu, Anne Njoki Gitere, Levi A. Muyela, Catherine Bikeri Onyancha, Litha Musili, Raechel Kamau, Alice Moraa Ondieki, Zul Merali, Bernard Alaka, Thomas Thesen, Mansoor Saleh, Adam P. Spira, Christine M Walsh, Omonigho M Bubu, Karen Blackmon, Chinedu Udeh‐Momoh

**Affiliations:** ^1^ Brain and Mind Institute, Aga Khan University, Nairobi, Kenya; ^2^ Geisel School of Medicine at Dartmouth, Hanover, NH, USA; ^3^ Clinical Research Unit, Aga Khan University, Nairobi, Kenya; ^4^ Department of Mental Health, Johns Hopkins Bloomberg School of Public Health, Baltimore, MD, USA; ^5^ Memory and Aging Center, UCSF Weill Institute for Neurosciences, University of California San Francisco, San Francisco, CA, USA; ^6^ Department of Neurology, NYU Grossman School of Medicine, New York, NY, USA; ^7^ Department of Psychiatry, NYU Grossman School of Medicine, New York, NY, USA; ^8^ Department of Population Health, NYU Grossman School of Medicine, New York, NY, USA; ^9^ Wake Forest University, School of Medicine, Winston‐Salem, NC, USA

## Abstract

**Background:**

Sleep disturbances are increasingly linked to **Alzheimer's disease (AD)**. Short sleep duration impairs memory whilst longer duration is linked to neuroinflammation and underlying neuropathology. Sleep health remains understudied in Africa, limiting insights into its role in dementia risk. Thus, we examined relationships between subjective sleep quality and memory complaints, focusing on dementia; and highlighting implications of self‐reported sleep in African populations.

**Method:**

Participants (*n* = 263; mean age 54.97; 60.1% female) from the Brain Resilience Kenya (BRK) and AD‐Detect Kenya studies were included and categorized into four groups: Dementia cases (*n* = 32, mean age 71.7), AD‐Detect and BRK Cognitively unimpaired controls (*n* = 201), and Oncology patients (breast/prostate cancer, *n* = 23). Self‐reported sleep was assessed using the 6‐item RU‐SATED questionnaire, measuring: Regularity; Uninterrupted Sleep; Satisfaction; Alertness; Timing; Efficiency; Duration (total score range: poor (0) – excellent (12)). Self‐reported cognitive concerns were compared against RU‐SATED score.

Logistic regression models were used to examine relationships between memory performance (binary outcome) and predictors across groups: RU‐SATED score, age, biological sex, education level and study group.

**Result:**

Dementia cases had the highest memory complaints (90.6%, *n* = 29) versus AD‐Detect controls (46.8%, *n* = 36), BRK controls (46.6%, *n* = 61), and oncology patients (47.8%, *n* = 11). Surprisingly, dementia cases recorded the highest mean RU‐SATED score (9.03, SD 1.94), followed by oncology patients (8.52, SD 2.84), AD‐Detect controls (7.71, SD 2.25), and BRK controls (7.49, SD 2.47).

No significant correlation emerged between self‐reported sleep quality and memory complaints. However, regression analysis (χ²(10) = 58.80, *p* < 0.001) identified age as a strong predictor of memory complaints (OR = 1.07, *p* < 0.001), with each additional year increasing complaint likelihood by 7%. Males were 56% less likely than females to report memory complaints (OR = 0.44, *p* = 0.005).

Sleep data measured via actigraphy will be presented.

**Conclusion:**

Preliminary findings suggest reporter bias in dementia patients with memory impairments, highlighting the need for gold‐standard objective ascertained sleep data. In LMICs like Kenya, where sleep health data is scarce, reliance on self‐reported assessments may introduce inaccuracies. Future studies should leverage objective measures (e.g., actigraphy, polysomnography) and informant verification to improve reliability, informing dementia risk reduction strategies in underrepresented populations.

